# 2-Benzyl-5-meth­oxy­isoindoline-1,3-dione

**DOI:** 10.1107/S160053681302638X

**Published:** 2013-09-28

**Authors:** Noemi Vila, María Carmen Costas-Lago, Pedro Besada, Carmen Terán

**Affiliations:** a Department of Organic Chemistry, University of Vigo, E-36310 Vigo, Spain

## Abstract

The title *N*-benzyl­phthalimide derivative, C_16_H_13_NO_3_, consists of two planar moieties, *viz.* the phthalimide system (r.m.s. deviation = 0.007 Å) and the phenyl ring, which make a dihedral angle of 84.7 (6)°. The meth­oxy group is almost coplanar with the phathalimide ring, as shown by the C—C—O—C torsion angle of −171.5 (2)°. In the crystal, the mol­ecules are self-assembled *via* non-classical C—H⋯O hydrogen bonds, forming a tape motif along [110].

## Related literature

For background to the applications of phthalimide derivatives, see: Luzzio (2005 ▶); Barooah & Baruah (2007 ▶); Sharma *et al.* (2010 ▶); Warzecha *et al.* (2006 ▶). For different approaches to synthesize *N*-benzyl­phthalimides, see: Luzzio (2005 ▶); Cao & Alper (2010 ▶); Vidal *et al.* (2000 ▶). For the synthesis of the title compound, see: Favor *et al.* (2008 ▶); Haj-Yehia & Khan (2004 ▶). For related structures, see: Warzecha *et al.* (2006*a* ▶,*b* ▶,*c* ▶); Jiang *et al.* (2008 ▶). 
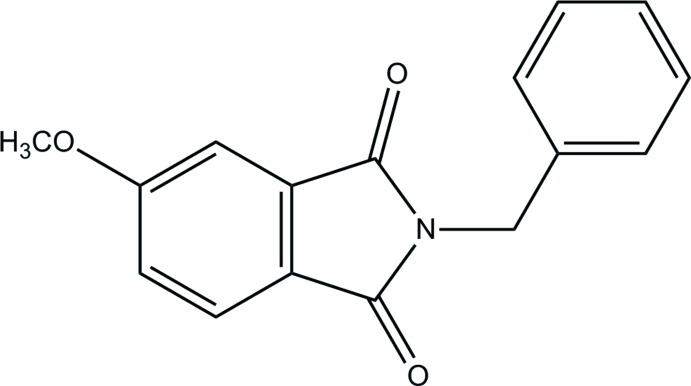


## Experimental

### 

#### Crystal data

C_16_H_13_NO_3_
*M* *_r_* = 267.27
Monoclinic, 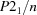

*a* = 8.476 (4) Å
*b* = 5.264 (3) Å
*c* = 28.295 (13) Å
β = 93.589 (9)°
*V* = 1260.0 (11) Å^3^
*Z* = 4
Mo *K*α radiation
μ = 0.10 mm^−1^
*T* = 100 K
0.49 × 0.13 × 0.07 mm

#### Data collection

Bruker SMART 1000 CCD diffractometer
Absorption correction: multi-scan (*SADABS*; Sheldrick, 1996 ▶) *T*_min_ = 0.954, *T*_max_ = 0.993
5768 measured reflections
2204 independent reflections
1433 reflections with *I* > 2σ(*I*)
*R*_int_ = 0.083

#### Refinement

*R*[*F* ^2^ > 2σ(*F* ^2^)] = 0.053
*wR*(*F* ^2^) = 0.137
*S* = 1.00
2204 reflections
183 parameters
H-atom parameters constrained
Δρ_max_ = 0.30 e Å^−3^
Δρ_min_ = −0.27 e Å^−3^

### 

Data collection: *SMART* (Bruker, 1998 ▶); cell refinement: *SAINT* (Bruker, 1998 ▶); data reduction: *SAINT*; program(s) used to solve structure: *SHELXS97* (Sheldrick, 2008 ▶); program(s) used to refine structure: *SHELXL97* (Sheldrick, 2008 ▶); molecular graphics: *PLATON* (Spek, 2009 ▶) and *Mercury* (Macrae *et al.*, 2006 ▶); software used to prepare material for publication: *SHELXTL* (Sheldrick, 2008 ▶).

## Supplementary Material

Crystal structure: contains datablock(s) I, New_Global_Publ_Block. DOI: 10.1107/S160053681302638X/fy2100sup1.cif

Structure factors: contains datablock(s) I. DOI: 10.1107/S160053681302638X/fy2100Isup2.hkl

Supplementary material file. DOI: 10.1107/S160053681302638X/fy2100Isup3.cml

Additional supplementary materials:  crystallographic information; 3D view; checkCIF report

## Figures and Tables

**Table 1:** Hydrogen-bond geometry (Å, °)

*D*—H⋯*A*	*D*—H	H⋯*A*	*D*⋯*A*	*D*—H⋯*A*
C4—H4⋯O5^i^	0.95	2.57	3.505 (3)	168
C7—H7⋯O1^ii^	0.95	2.40	3.247 (3)	149
C8—H8*B*⋯O3^i^	0.98	2.59	3.432 (4)	144

Symmetry codes: (i) 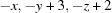; (ii) 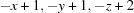.

**Fig. 1.**
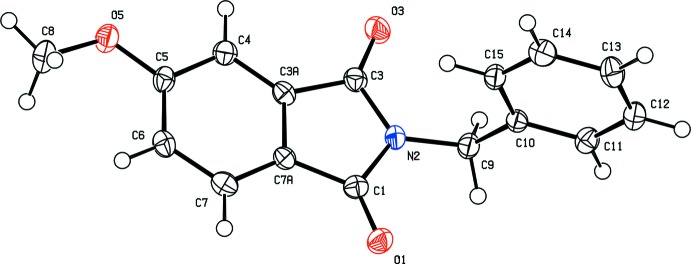
Molecular structure of (I) showing the atom-numbering scheme. Displacement ellipsoids are shown at the 50% probability level.

**Fig. 2.**
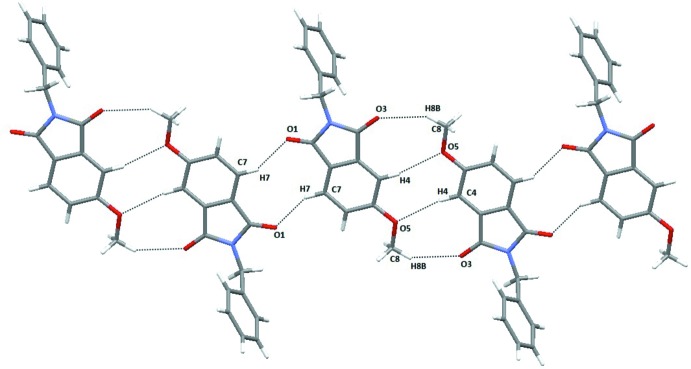
View of the supramolecular tape motif in the crystal structure of the title compound.

**Fig. 3.**
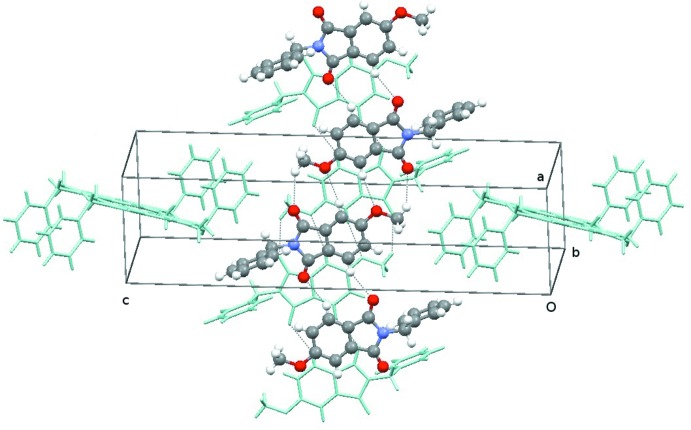
View of the unit-cell contents of (I).

## supplementary crystallographic information

### 1. Comment

Phthalimide derivatives represent a significant family of organic compounds because of their numerous applications in different fields of chemistry. They are not only useful intermediates for synthesis (Luzzio, 2005), but are also important scaffolds for new materials (Barooah & Baruah, 2007) and drug design (Sharma *et al.*, 2010). Among the phthalimide analogues, there are very well known *N*-benzyl substituted derivatives, some of them prepared for mechanistic studies on photoreactions (Warzecha, Görner *et al.*, 2006). Reaction of phthalic acid derivatives with benzylamines at high temperature or in the presence of a Lewis acid are the classical methods for obtaining *N*-benzylphthalimides (Favor *et al.*, 2008; Haj-Yehia & Khan, 2004; Luzzio, 2005). In addition, unconventional approaches were also developed, such as carbonylative cyclizations of arenes with amines catalyzed by transition metals (Cao & Alper, 2010) or microwave-assisted synthesis (Vidal *et al.*, 2000).

The title compound (I) is a *N*-benzylphthalimide substituted at C5 with a methoxy group. It was obtained by the reaction of dimethyl phthalimide with benzyl hydrazine under microwave irradiation.

The molecular structure of (I) is illustrated in Figure 1. There are some similar structures reported before (Warzecha *et al.*, 2006*a*; Warzecha *et al.*, 2006*b*; Warzecha *et al.*, 2006*c*; Jiang *et al.*, 2008). The molecule consists of two planar moieties, the phthalimide system and the phenyl ring, linked by the methylene group C9 (N2—C9—C10 bond angle of 114.4°), resulting in a non-planar structure. The two planar subunits make a dihedral angle of 84.7 (6)°, which is similar to the value reported for the same angle in the crystal structure of the monoclinic form of the parent *N*-benzylphthalimide (Jiang *et al.*, 2008). Furthermore, the methoxy group at C5 is almost coplanar with the phathalimide ring [torsion angle C4—C5—O5—C8 of -171.5 (2)°].

In addition, the C1—N2—C9—C10 and C3—N2—C9—C10 torsion angles of 93.1 (3)° and -86.0 (3)°, respectively, are also very similar to those of *N*-benzylphthalimide (Jiang *et al.*, 2008) and they corroborate that the phenyl group is virtually orthogonal to the phthalimide benzene ring. In the crystal structure, the molecules are self-assembled *via* non-classical C—H···O hydrogen bonds, involving CH and CH_3_ groups as donors and oxygen atoms as acceptors, to form a one-dimensional supramolecular organization (Table 1, Figure 2).

### 2. Experimental

The synthesis of 2-benzyl-5-methoxyisoindoline-1,3-dione was carried out in a microwave oven (CEM discover system 908010, monomode) according to the following protocol: a solution of dimethyl 4-methoxyphthalate (50 mg, 0.22 mmol), benzylhydrazine dihydrochloride (174 mg, 0.89 mmol) and triethylamine (0.37 ml, 2.65 mmol), in ethanol (5 ml) was introduced in a Pyrex flask and submitted to microwave irradiation (280 W, 185 °C) for 30 min. The solvent was evaporated under reduced pressure and the residue was purified by column chromatography on silica gel (hexane/ethyl acetate 40:1 → 20:1) to afford a white solid (9.3 mg, 15%). The product was dissolved in ethyl acetate (3 ml) and the solution was kept at room temperature for 1 d. Natural evaporation gave colourless block-like crystals of the title compound (m.p. 448–449 K) suitable for X-ray diffraction analysis.

### 3. Refinement

All H-atoms were positioned and refined using a riding model with d(C—H)= 0.95 Å, *U*_iso_ = 1.2*U*_eq_(C) for aromatic CH, d(C—H)= 0.99 Å, *U*_iso_ = 1.2*U*_eq_(C) for CH_2_ group and d(C—H)= 0.98 Å, *U*_iso_ = 1.5*U*_eq_(C) for CH_3_ group.

### Crystal data

**Table:**

C_16_H_13_NO_3_	*F*(000) = 560
*M**_r_* = 267.27	*D*_x_ = 1.409 Mg m^−^^3^
Monoclinic, *P*2_1_/*n*	Mo *K*α radiation, λ = 0.71073 Å
*a* = 8.476 (4) Å	Cell parameters from 1091 reflections
*b* = 5.264 (3) Å	θ = 2.6–24.8°
*c* = 28.295 (13) Å	µ = 0.10 mm^−^^1^
β = 93.589 (9)°	*T* = 100 K
*V* = 1260.0 (11) Å^3^	Prism, colourless
*Z* = 4	0.49 × 0.13 × 0.07 mm

### Data collection

**Table:**

Bruker SMART 1000 CCD diffractometer	2204 independent reflections
Radiation source: fine-focus sealed tube	1433 reflections with *I* > 2σ(*I*)
Graphite monochromator	*R*_int_ = 0.083
φ and ω scans	θ_max_ = 25.1°, θ_min_ = 1.4°
Absorption correction: multi-scan (*SADABS*; Sheldrick, 1996)	*h* = −10→9
*T*_min_ = 0.954, *T*_max_ = 0.993	*k* = −5→6
5768 measured reflections	*l* = −33→31

### Refinement

**Table:**

Refinement on *F*^2^	Secondary atom site location: difference Fourier map
Least-squares matrix: full	Hydrogen site location: inferred from neighbouring sites
*R*[*F*^2^ > 2σ(*F*^2^)] = 0.053	H-atom parameters constrained
*wR*(*F*^2^) = 0.137	*w* = 1/[σ^2^(*F*_o_^2^) + (0.0548*P*)^2^] where *P* = (*F*_o_^2^ + 2*F*_c_^2^)/3
*S* = 1.00	(Δ/σ)_max_ < 0.001
2204 reflections	Δρ_max_ = 0.30 e Å^−^^3^
183 parameters	Δρ_min_ = −0.27 e Å^−^^3^
0 restraints	Extinction correction: *SHELXL97* (Sheldrick, 2008), Fc^*^=kFc[1+0.001xFc^2^λ^3^/sin(2θ)]^-1/4^
Primary atom site location: structure-invariant direct methods	Extinction coefficient: 0.022 (4)

### Special details

**Table:**

Experimental. ^1^H NMR (400 MHz, CDCl_3_) δ p.p.m.: 7.77 (d, *J* = 8.3 Hz, 1H, H7), 7.44 (m, 2H, H—Ph), 7.31 (m, 4H, H4, 3xH-Ph), 7.16 (dd, *J* = 8.3 Hz, 2.3 Hz, 1H, H6), 4.85 (s, 2H, CH_2_), 3.95 (s, 3H, OCH_3_). ^13^C MNR (100 MHz, CDCl_3_) δ p.p.m.: 167.9 (2xCO), 164.7 (C5), 136.5 (C), 134.7 (C), 128.7 (CH—Ar), 128.6 (CH—Ar), 127.8 (CH—Ar), 125.1 (C7), 124.0 (C), 119.7 (C6), 108.2 (C4), 56.1 (CH_3_), 41.6 (CH_2_). EMAR (ESI) calcld. for: [C_16_H_14_NO_3_]^+^ 268.09682; Found: 268.09697
Geometry. All e.s.d.'s (except the e.s.d. in the dihedral angle between two l.s. planes) are estimated using the full covariance matrix. The cell e.s.d.'s are taken into account individually in the estimation of e.s.d.'s in distances, angles and torsion angles; correlations between e.s.d.'s in cell parameters are only used when they are defined by crystal symmetry. An approximate (isotropic) treatment of cell e.s.d.'s is used for estimating e.s.d.'s involving l.s. planes.
Refinement. Refinement of *F*^2^ against ALL reflections. The weighted *R*-factor *wR* and goodness of fit *S* are based on *F*^2^, conventional *R*-factors *R* are based on *F*, with *F* set to zero for negative *F*^2^. The threshold expression of *F*^2^ > σ(*F*^2^) is used only for calculating *R*-factors(gt) *etc*. and is not relevant to the choice of reflections for refinement. *R*-factors based on *F*^2^ are statistically about twice as large as those based on *F*, and *R*- factors based on ALL data will be even larger.

### Fractional atomic coordinates and isotropic or equivalent isotropic displacement parameters (Å^2^)

**Table:**

	*x*	*y*	*z*	*U*_iso_*/*U*_eq_	
C1	0.3240 (3)	0.6996 (5)	0.92388 (10)	0.0213 (6)	
O1	0.4087 (2)	0.5180 (4)	0.91838 (6)	0.0267 (5)	
N2	0.2352 (3)	0.8192 (4)	0.88663 (7)	0.0211 (6)	
C3	0.1503 (3)	1.0257 (5)	0.90228 (10)	0.0215 (7)	
O3	0.0646 (2)	1.1594 (4)	0.87683 (7)	0.0288 (5)	
C3A	0.1874 (3)	1.0402 (5)	0.95430 (9)	0.0195 (6)	
C4	0.1349 (3)	1.2090 (5)	0.98669 (10)	0.0231 (7)	
H4	0.0649	1.3435	0.9774	0.028*	
C5	0.1887 (3)	1.1749 (5)	1.03403 (9)	0.0217 (6)	
O5	0.1292 (2)	1.3429 (4)	1.06478 (6)	0.0270 (5)	
C6	0.2935 (3)	0.9800 (5)	1.04724 (10)	0.0230 (7)	
H6	0.3303	0.9626	1.0795	0.028*	
C7	0.3447 (3)	0.8103 (5)	1.01369 (10)	0.0241 (7)	
H7	0.4147	0.6754	1.0227	0.029*	
C7A	0.2915 (3)	0.8428 (5)	0.96708 (9)	0.0197 (6)	
C8	0.1941 (3)	1.3450 (6)	1.11298 (10)	0.0310 (8)	
H8A	0.1716	1.1825	1.1281	0.047*	
H8B	0.1462	1.4834	1.1303	0.047*	
H8C	0.3087	1.3704	1.1134	0.047*	
C9	0.2351 (3)	0.7371 (5)	0.83788 (9)	0.0232 (7)	
H9A	0.1327	0.7845	0.8215	0.028*	
H9B	0.2432	0.5495	0.8372	0.028*	
C10	0.3673 (3)	0.8478 (5)	0.81074 (9)	0.0195 (6)	
C11	0.3994 (3)	0.7401 (5)	0.76729 (9)	0.0232 (7)	
H11	0.3424	0.5937	0.7565	0.028*	
C12	0.5122 (3)	0.8419 (6)	0.73974 (10)	0.0253 (7)	
H12	0.5313	0.7673	0.7101	0.030*	
C13	0.5972 (3)	1.0524 (6)	0.75539 (10)	0.0281 (7)	
H13	0.6753	1.1231	0.7366	0.034*	
C14	0.5679 (3)	1.1605 (5)	0.79876 (10)	0.0277 (7)	
H14	0.6267	1.3050	0.8096	0.033*	
C15	0.4534 (3)	1.0593 (5)	0.82639 (10)	0.0226 (7)	
H15	0.4340	1.1348	0.8560	0.027*	

### Atomic displacement parameters (Å^2^)

**Table:**

	*U*^11^	*U*^22^	*U*^33^	*U*^12^	*U*^13^	*U*^23^
C1	0.0199 (14)	0.0202 (15)	0.0238 (16)	−0.0009 (12)	0.0017 (12)	0.0003 (12)
O1	0.0280 (11)	0.0264 (11)	0.0255 (11)	0.0068 (10)	0.0011 (9)	−0.0021 (9)
N2	0.0249 (13)	0.0229 (13)	0.0156 (12)	0.0022 (10)	0.0021 (10)	−0.0003 (10)
C3	0.0205 (14)	0.0225 (15)	0.0219 (15)	0.0000 (13)	0.0047 (12)	0.0022 (12)
O3	0.0307 (11)	0.0333 (12)	0.0222 (11)	0.0097 (9)	−0.0004 (9)	0.0040 (9)
C3A	0.0157 (13)	0.0227 (15)	0.0201 (15)	−0.0023 (11)	0.0027 (11)	0.0035 (12)
C4	0.0216 (14)	0.0237 (15)	0.0245 (16)	0.0014 (12)	0.0058 (12)	0.0017 (12)
C5	0.0202 (14)	0.0248 (15)	0.0207 (15)	−0.0026 (13)	0.0059 (12)	−0.0024 (13)
O5	0.0278 (11)	0.0317 (11)	0.0213 (11)	0.0052 (9)	0.0000 (9)	−0.0049 (9)
C6	0.0237 (15)	0.0268 (16)	0.0182 (15)	−0.0041 (13)	−0.0001 (12)	0.0008 (13)
C7	0.0224 (15)	0.0241 (15)	0.0257 (16)	−0.0017 (12)	0.0001 (12)	0.0039 (13)
C7A	0.0205 (14)	0.0192 (14)	0.0197 (15)	−0.0022 (12)	0.0043 (12)	0.0020 (12)
C8	0.0300 (17)	0.0402 (19)	0.0231 (16)	0.0028 (14)	0.0029 (13)	−0.0070 (14)
C9	0.0256 (16)	0.0252 (16)	0.0190 (15)	0.0025 (12)	0.0017 (12)	−0.0026 (12)
C10	0.0193 (14)	0.0223 (15)	0.0167 (14)	0.0054 (12)	−0.0008 (11)	0.0005 (12)
C11	0.0213 (15)	0.0257 (16)	0.0223 (16)	0.0008 (12)	−0.0014 (13)	−0.0036 (12)
C12	0.0244 (15)	0.0313 (17)	0.0202 (15)	0.0030 (13)	0.0019 (12)	−0.0031 (13)
C13	0.0240 (15)	0.0374 (18)	0.0232 (16)	−0.0008 (14)	0.0044 (13)	0.0011 (14)
C14	0.0279 (16)	0.0269 (16)	0.0279 (17)	−0.0027 (13)	−0.0026 (13)	0.0001 (14)
C15	0.0247 (15)	0.0254 (16)	0.0177 (15)	0.0034 (13)	0.0015 (12)	−0.0017 (12)

### Geometric parameters (Å, º)

**Table:**

C1—O1	1.211 (3)	C8—H8A	0.9800
C1—N2	1.405 (3)	C8—H8B	0.9800
C1—C7A	1.476 (4)	C8—H8C	0.9800
N2—C3	1.391 (3)	C9—C10	1.514 (4)
N2—C9	1.445 (3)	C9—H9A	0.9900
C3—O3	1.215 (3)	C9—H9B	0.9900
C3—C3A	1.488 (4)	C10—C15	1.388 (4)
C3A—C4	1.371 (4)	C10—C11	1.396 (4)
C3A—C7A	1.396 (4)	C11—C12	1.379 (4)
C4—C5	1.399 (4)	C11—H11	0.9500
C4—H4	0.9500	C12—C13	1.379 (4)
C5—O5	1.359 (3)	C12—H12	0.9500
C5—C6	1.393 (4)	C13—C14	1.389 (4)
O5—C8	1.438 (3)	C13—H13	0.9500
C6—C7	1.393 (4)	C14—C15	1.390 (4)
C6—H6	0.9500	C14—H14	0.9500
C7—C7A	1.378 (4)	C15—H15	0.9500
C7—H7	0.9500		
			
O1—C1—N2	123.4 (2)	O5—C8—H8B	109.5
O1—C1—C7A	130.7 (2)	H8A—C8—H8B	109.5
N2—C1—C7A	105.9 (2)	O5—C8—H8C	109.5
C3—N2—C1	112.0 (2)	H8A—C8—H8C	109.5
C3—N2—C9	124.6 (2)	H8B—C8—H8C	109.5
C1—N2—C9	123.4 (2)	N2—C9—C10	114.4 (2)
O3—C3—N2	124.5 (3)	N2—C9—H9A	108.7
O3—C3—C3A	129.7 (3)	C10—C9—H9A	108.7
N2—C3—C3A	105.9 (2)	N2—C9—H9B	108.7
C4—C3A—C7A	122.4 (2)	C10—C9—H9B	108.7
C4—C3A—C3	129.6 (2)	H9A—C9—H9B	107.6
C7A—C3A—C3	108.0 (2)	C15—C10—C11	118.6 (3)
C3A—C4—C5	117.2 (2)	C15—C10—C9	122.5 (2)
C3A—C4—H4	121.4	C11—C10—C9	118.8 (2)
C5—C4—H4	121.4	C12—C11—C10	121.3 (3)
O5—C5—C6	124.3 (2)	C12—C11—H11	119.3
O5—C5—C4	114.7 (2)	C10—C11—H11	119.3
C6—C5—C4	121.0 (3)	C11—C12—C13	119.8 (3)
C5—O5—C8	118.5 (2)	C11—C12—H12	120.1
C5—C6—C7	120.7 (2)	C13—C12—H12	120.1
C5—C6—H6	119.6	C12—C13—C14	119.6 (3)
C7—C6—H6	119.6	C12—C13—H13	120.2
C7A—C7—C6	118.4 (3)	C14—C13—H13	120.2
C7A—C7—H7	120.8	C13—C14—C15	120.6 (3)
C6—C7—H7	120.8	C13—C14—H14	119.7
C7—C7A—C3A	120.3 (3)	C15—C14—H14	119.7
C7—C7A—C1	131.5 (2)	C10—C15—C14	120.0 (3)
C3A—C7A—C1	108.3 (2)	C10—C15—H15	120.0
O5—C8—H8A	109.5	C14—C15—H15	120.0
			
O1—C1—N2—C3	−178.9 (3)	C6—C7—C7A—C1	179.5 (3)
C7A—C1—N2—C3	0.3 (3)	C4—C3A—C7A—C7	0.5 (4)
O1—C1—N2—C9	0.3 (4)	C3—C3A—C7A—C7	−179.5 (2)
C7A—C1—N2—C9	179.5 (2)	C4—C3A—C7A—C1	−179.7 (2)
C1—N2—C3—O3	−179.9 (3)	C3—C3A—C7A—C1	0.3 (3)
C9—N2—C3—O3	0.9 (4)	O1—C1—C7A—C7	−1.4 (5)
C1—N2—C3—C3A	−0.1 (3)	N2—C1—C7A—C7	179.4 (3)
C9—N2—C3—C3A	−179.3 (2)	O1—C1—C7A—C3A	178.7 (3)
O3—C3—C3A—C4	−0.3 (5)	N2—C1—C7A—C3A	−0.4 (3)
N2—C3—C3A—C4	179.9 (3)	C3—N2—C9—C10	93.1 (3)
O3—C3—C3A—C7A	179.6 (3)	C1—N2—C9—C10	−86.0 (3)
N2—C3—C3A—C7A	−0.1 (3)	N2—C9—C10—C15	−16.9 (3)
C7A—C3A—C4—C5	−0.7 (4)	N2—C9—C10—C11	166.0 (2)
C3—C3A—C4—C5	179.3 (3)	C15—C10—C11—C12	−1.0 (4)
C3A—C4—C5—O5	−178.1 (2)	C9—C10—C11—C12	176.2 (2)
C3A—C4—C5—C6	1.1 (4)	C10—C11—C12—C13	0.9 (4)
C6—C5—O5—C8	9.2 (4)	C11—C12—C13—C14	−0.2 (4)
C4—C5—O5—C8	−171.5 (2)	C12—C13—C14—C15	−0.3 (4)
O5—C5—C6—C7	177.8 (2)	C11—C10—C15—C14	0.5 (4)
C4—C5—C6—C7	−1.4 (4)	C9—C10—C15—C14	−176.6 (2)
C5—C6—C7—C7A	1.1 (4)	C13—C14—C15—C10	0.1 (4)
C6—C7—C7A—C3A	−0.7 (4)		

### Hydrogen-bond geometry (Å, º)

**Table:**

*D*—H···*A*	*D*—H	H···*A*	*D*···*A*	*D*—H···*A*
C4—H4···O5^i^	0.95	2.57	3.505 (3)	168
C7—H7···O1^ii^	0.95	2.40	3.247 (3)	149
C8—H8*B*···O3^i^	0.98	2.59	3.432 (4)	144
C15—H15···N2	0.95	2.56	2.884 (4)	100

Symmetry codes: (i) −*x*, −*y*+3, −*z*+2; (ii) −*x*+1, −*y*+1, −*z*+2.
